# Drug dosing in the critically ill obese patient—a focus on sedation, analgesia, and delirium

**DOI:** 10.1186/s13054-020-03040-z

**Published:** 2020-06-08

**Authors:** Brian L. Erstad, Jeffrey F. Barletta

**Affiliations:** 1 Department of Pharmacy Practice & Science, University of Arizona, College of Pharmacy, 1295 N Martin Ave, PO Box 210202, Tucson, AZ 85721 USA; 2 Department of Pharmacy Practice, Midwestern University, College of Pharmacy, 19555 N 59th Ave, Glendale, AZ 85308 USA

**Keywords:** Critical illness, Obesity, Pharmacokinetics, Drug-dosing

## Abstract

Practice guidelines provide clear evidence-based recommendations for the use of drug therapy to manage pain, agitation, and delirium associated with critical illness. Dosing recommendations however are often based on strategies used in patients with normal body habitus. Recommendations specific to critically ill patients with extreme obesity are lacking. Nonetheless, clinicians must craft dosing regimens for this population. This paper is intended to help clinicians design initial dosing regimens for medications commonly used in the management of pain, agitation, and delirium in critically ill patients with extreme obesity. A detailed literature search was conducted with an emphasis on obesity, pharmacokinetics, and dosing. Relevant manuscripts were reviewed and strategies for dosing are provided.

## Introduction

Only 14 of the 100 most commonly used injectable medications in the adult intensive care units (ICUs) of an academic medical center had information in product labeling related to dosing obese patients. Of these 14 medications, only 6 had information considered to be minimally adequate for dosing [1]. Further, recommendations for medication dosing in critically ill obese patients are not available from adequately powered randomized studies with clinically relevant endpoints [2]. Prospective studies that are available typically used single-dose injections of medication in small numbers of patients in non-ICU settings. Additionally, available studies involving drug dosing in obese patients often evaluated pharmacokinetic parameters and/or surrogate markers as indicators of efficacy and adverse effects rather than clinically important outcomes. Regardless of suboptimal information, clinicians have to devise dosing regimens for critically ill obese patients that account for patient-specific issues. Therefore, dosing recommendations for patients with obesity usually are extrapolated from the physicochemical characteristics of medications in conjunction with data from pharmacokinetic, pharmacodynamic, and retrospective studies. The purpose of this paper is to provide a framework to help clinicians design initial dosing regimens for medications commonly used for the management of sedation, analgesia, and delirium in critically ill obese patients as discussed in evidence-based guidelines [3]. The focus will be on adult patients with more severe forms of obesity (e.g., body mass index (BMI) ≥ 40 kg/m^2^), since such patients are typically limited in numbers in the studies used to formulate product labeling information and therefore most likely to receive inappropriate dosing if conventional doses are employed.

## Methodology

A detailed literature search was performed using PubMed from inception to October 2019, using search terms from the following three categories: (1)obesity: “Obesity”[Mesh] OR “Overweight”[Mesh] OR “body composition”[MeSH Terms] OR “body weight change*” OR “body size” OR “extreme obesity” OR “body fat” OR “body fatness,” (2) pharmacokinetics and dosing: “Drug Monitoring”[Mesh] OR “Dose-Response Relationship, Drug”[Mesh] OR “pharmacokinetic considerations” OR “pharmacokinetic” OR “therapeutic drug monitoring” OR “drug monitoring” OR “drug dosing” OR “drug dose,” and (3) the specific drug in question. The medications reviewed were those listed in recommendations from evidence-based guidelines and include the following: opioids, non-opioid analgesics, ketamine, propofol, dexmedetomidine, benzodiazepines, etomidate, haloperidol, and quetiapine. Results from the primary literature search were reviewed and pertinent articles were retained. Bibliographies were reviewed for articles that may have been missed by the primary literature search. Non-English articles and animal studies were not included. A comprehensive, online database was consulted for drug physicochemical properties (e.g., log *P*) [4]. Suggestions were then formed using the available data based on the following prioritization strategy: outcome-based studies, pharmacokinetic studies, adverse effect profiles, and physiochemical properties. Because of the heterogeneity of study outcomes (i.e., pharmacokinetic-related, and clinical outcome) and the expected lack of information for many of the medications included, advanced statistical techniques such as meta-analysis was not performed.

When designing strategies for analgesia, sedation, or delirium, recommendations from evidence-based guidelines should be adhered to, similar to non-obese patients [3]. This includes targeting light sedation, use of validated assessment scales, daily sedative interruptions, spontaneous breathing trials, and delirium screening. When dosing medications, the choice of weight descriptor might seem to make little difference for weight-based dosing regimens because medications are dosed to clinical effect. However, particularly during medication initiation, there is the potential for under- or over-dosing depending on the choice of weight descriptor. For example, the recommended starting rate of a propofol infusion for ICU sedation is 5 mcg/kg/min, as recommended in product information based on the clinical trials leading to drug approval, which likely involved normal weight individuals. Three critically ill patients all of the same age, height, sex, and co-morbidities, but weighing 70 , 100, and 140 kg, would receive starting doses ranging from 350 to 700 mcg/min based on ABW. (Additional file 1) While propofol (and other medications described in this paper) can be titrated to effect, large initial doses or infusion rates (based on ABW) can lead to adverse cardiovascular consequences, attainment of deeper levels of sedation, or doses that exceed those recommended for safety. Furthermore, the occurrence of an adverse drug event can lead to premature discontinuation of therapy and substitution with a less attractive alternative (i.e., benzodiazepine infusion). On the other extreme, under-dosing is a concern if ideal body weight (IBW) is inappropriately used for weight-based dosing regimens, since the calculation for IBW only considers height and not excess weight. This is particularly relevant for patients who receive etomidate prior to neuromuscular blocker therapy as under-dosing may lead an awake state during paralysis [5].

Several alternative size descriptors to actual body weight (ABW) exist for weight-based dosing because it is known that lean mass does not increase in proportion to fat mass in obese subjects [6]. Ideal body weight only considers height and gender and thought to have originated from life insurance tables published more than 60 years ago. Unless otherwise stated, IBW refers to 50 kg + 2.3 kg per inch above 60 in. for men or 45.5 kg + 2.3 kg per inch above 60 in. for women. Adjusted body weight refers to IBW kg + 0.4 (ABW kg – IBW kg) for men or women. Body surface area (BSA) is considered the gold standard for dosing many chemotherapeutic medications and BMI is considered the gold standard for assessing obesity. Both however have limitations as size descriptors for medication dosing [7]. Lean body mass and allometric scaling are other size descriptors being investigated for medication dosing. More recent investigations evaluating lean body mass for medication dosing use separate equations for men and women and are susceptible to calculation errors if not calculated by software programs [8]. Further, equations to estimate lean body mass are not reliable in critically ill patients when compared to computed tomography as the gold standard [9]. Allometric scaling is a much simpler equation used to predict volume of distribution and clearance parameters from animals to humans and is currently being studied for its predictive value from normal weight to obese subjects [10].

**Fig. 1:**
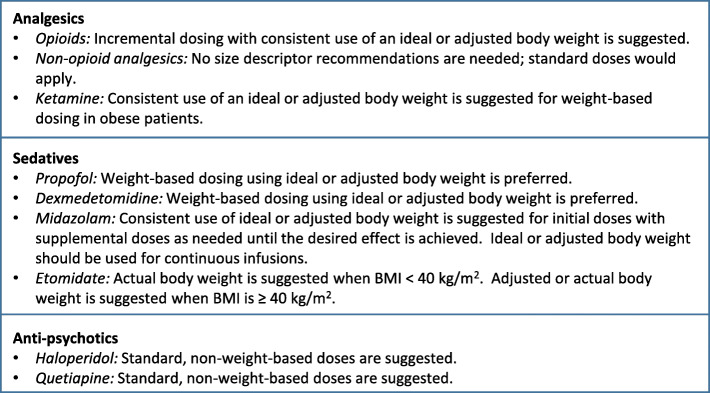
Summary of dosing recommendations for medications used in the management of sedation, analgesia, and delirium in critically ill patients with obesity. Unless otherwise indicated, these recommendations apply for patients with more severe forms of obesity (i.e., BMI ≥ 40 kg/m^2^)

Several assumptions or considerations underlie the recommendations in this paper. First, recommendations are limited to dosing based on ABW, or IBW or adjusted body weight equations recognizing the known limitations of each of these dosing descriptors [11]. For the purposes of this paper, adjusted body weight is considered to be roughly equivalent to lean body mass. The IBW and adjusted body equations have the advantages of being simple to calculate and well-known to most clinicians. While other size descriptors have theoretical appeal, none has demonstrated advantages for dosing obese patients based on clinical outcomes. Next, recorded ABW measurements assume accuracy (e.g., within 5% of ABW in kilograms) in weight measurements and consistent use of whatever weight descriptor (IBW, adjusted, actual) is chosen for dosing [12]. The recommendations in this paper also assume that other patient-specific considerations such as end-organ dysfunction (with impaired parent drug or active metabolite elimination) or actionable pharmacogenetic/pharmacogenomics profiles are considered in the final dosing recommendation. Finally, it should be noted that relatively few patients of extreme body weight served as the basis for dosing recommendations available in product labeling. A summary of these recommendations is listed in Fig. 1.

### Analgesia

#### Opioids

All opioids are lipophilic with log *P* values between 1 and 2 for morphine, hydromorphone, and remifentanil and above 3 for fentanyl and sufentanil. This suggests that ABW might be preferred for single weight-based doses of fentanyl and sufentanil, in particular, given the expected increasing volume of distribution with increasing body weight. However, in studies suggesting a size descriptor for dosing opioids, recommendations were for IBW, lean body mass, or adjusted body weight as a preferred descriptor. While no prospective studies are available that compare weight-based dosing of opioids in obese and normal weight critically ill patients, prospective and retrospective studies performed in the emergency department and postoperative setting have consistently found large variations in opioid requirements and pain control in overweight and obese patients that had no relationship to ABW [13–16]. Similarly, pharmacokinetic studies evaluating various opioids in the perioperative setting have found opioid doses based on ABW are likely to be excessive as evidenced by pharmacokinetic parameters and measured opioid concentrations [17–22]. One study noted decreased clearance of active morphine metabolites in morbidly obese compared to normal weight healthy volunteers, despite normal elimination of the parent compound. The clinical importance of this finding is unknown but could be of consequence with sustained dosing [23]. Pharmacodynamic alterations such as increased opioid sensitivity have also been described in select populations (e.g., geriatrics, obstructive sleep apnea) which can further increase the risk for respiratory depression [24, 25]. Guidelines promulgated by anesthesia associations in Great Britain and Ireland recommend the use of lean body weight for dosing opioids given the poor correlation between opioid concentrations and clinical effect and concerns related to overdosing until patients are alert allowing for dose titration [26]. Given the substantial variation in opioid pharmacokinetic parameters and clinical effects that are not related to ABW, doses titrated to effect are recommended regardless of whether weight-based or non-weight-based dosing is used.

##### Summary

For dosing opioids, incremental dosing titrated to clinical effect with consistent use of an ideal or adjusted body weight is suggested for weight-based dosing in obese patients, particularly in patients with more severe forms of obesity (e.g., BMI ≥ 40 kg/m^2^).

#### Non-opioid analgesics

Non-opioid analgesics commonly administered to critically ill patients typically use non-weight-based dosing regimens based on information in product literature given the lack of prospective studies evaluating weight-based regimens. The few pharmacokinetic and pharmacodynamic studies evaluating the disposition of non-opioid agents, such as nonsteroidal anti-inflammatory drugs and acetaminophen, suggest variable pharmacokinetic parameters with little benefit for dose individualization based on weight. Furthermore, there are adverse effect concerns when increasing doses beyond those needed to reach the analgesic ceiling effect [27–29]. Some adverse effects (e.g., acetaminophen-induced liver injury) may be more frequent in patients with obesity [30, 31]. Therefore, no size descriptor recommendation is needed for analgesic agents administered by non-weight-based dosing regimens.

#### Ketamine

Ketamine dosing varies based on the clinical indication with lower doses being utilized for the provision of analgosedation in mechanically ventilated patients (versus status epilepticus) [32]. Even within studies specific to this indication, there is wide divergence in dosing strategies and obese patients are not well represented. Thus, dosing recommendations in patients with more severe forms of obesity are dependent on extrapolations from ketamine’s physicochemical and pharmacokinetic characteristics. Substantial lipophilicity (log P 3.1) with a large volume of distribution, rapid clearance (approximately 18 mL/min/kg) approximating hepatic blood flow, and active metabolites all complicate potential dosing recommendations for ketamine, particularly with sustained dosing in critically ill patients. Studies in animals and healthy volunteers have demonstrated that ketamine has a large steady state volume of distribution of approximately 2.5 to 5 L/kg with estimates of initial (alpha) distribution half-life ranging from 2 to 20 min and terminal half-life ranging from 2.5 to 3 h [33, 34]. A study in critically ill patients with brain or spinal cord injuries found increases in ketamine’s volume of distribution and clearance. The increase in volume of distribution was more than three times that found in studies involving normal healthy volunteers or patients undergoing surgery. Clearance was also increased in critically ill patients, but less so than the volume of distribution, resulting in a terminal half-life of approximately 5 h [35]. With single or isolated doses, the alpha half-life corresponds to termination of analgesic (low dose) or anesthetic (high dose) actions of ketamine by redistribution from the CNS to peripheral tissues. However, with sustained intermittent intravenous injections or continuous infusions of ketamine, accumulation of both parent drug and active metabolite norketamine occurs until steady state conditions occur. Norketamine has not only about one-third the potency of the parent compound, but also has slower elimination that increases the time to reach steady state. Therefore, ketamine administered by continuous intravenous infusion will likely need decreases in dose over time in order to maintain the same clinical effect.

With respect to loading doses, ABW is appealing as a size descriptor given the lipophilicity of ketamine, since clinical effect in this situation is largely a function of the drug’s volume of distribution. This belies the substantial inter-patient variability and therefore predictability of volume of distribution in a critically ill patient. Further, clearance of ketamine, which becomes more of an issue with sustained dosing, is a function of lean body mass and not likely to increase in proportion to fat mass in patients with obesity. These factors in conjunction with the challenges related to dosing a drug with an active metabolite suggest the use of an ideal or adjusted body weight is preferable for weight-based dosing calculations due to adverse effect concerns associated with over-dosing.

##### Summary

For dosing ketamine, consistent use of an ideal or adjusted body weight is suggested for weight-based dosing in obese patients, particularly in patients with more severe forms of obesity (e.g., BMI ≥ 40 kg/m^2^).

### Sedation

#### Propofol

Propofol is one of the most widely used sedatives for the facilitation of mechanical ventilation because of its quick onset and short duration of effect. Propofol has a large volume of distribution (60 L/kg) with a log *P* of 4.16 indicating a high degree of lipophilicity. Several studies have evaluated propofol-dosing strategies in the operating room but there are no data specific to the ICU. Thus, when extrapolating these data to the ICU setting, differences in therapeutics goals, administration techniques, and treatment duration must be considered. One study evaluated propofol for maintenance of anesthesia in a cohort of patients with a mean weight of 115 ± 21 kg [36]. In this study, both clearance and volume of distribution were significantly correlated with weight (*r* = 0.76 and 0.69, respectively) indicating ABW may be the most suitable weight measure for maintenance of anesthesia. A second study described the relationship between propofol concentrations and weight and concluded plasma concentrations may be dependent on ABW (*r* = 0.646, *p* < .001) [37]. This study however included primarily non-obese individuals (ABW = 58 ± 13 kg) and patients with more extreme forms of obesity were not represented.

Other studies evaluating propofol for anesthesia induction have suggested alternatives such as lean body weight or corrected body weight as the preferred metric for dosing secondary to the nonlinear relationship that exists in obese patients between ABW and clearance (Additional file 2) [38–45]. One study described a pharmacokinetic model in obesity that replaced ABW with lean body weight and simulated concentrations were higher with ABW-based dosing [43]. A second study randomized obese patients (BMI > 35 kg/m^2^) receiving propofol for anesthesia induction based on either ABW or lean body weight-based dosing; pharmacokinetic and pharmacodynamic effects were then compared to a cohort of non-obese patients (BMI < 25 kg/m^2^) [39]. In this study, both clearance (lean body weight-dosing, 9.15 L/min, ABW-dosing 10 L/min versus 4.11 L/min, *p* < .01) and volume of the peripheral compartment (lean body weight-dosing, 84.2 L, ABW-dosing, 73.2 L versus 46.9 L, *p* < .01) were greater in the patients with obesity. Patients who were dosed based on ABW, however, had significantly lower bispectral index values indicating a heightened anesthetic response and increased CNS sensitivity. Dosing using lean body weight was therefore recommended.

Other studies have accounted for the non-linear relationship between weight and clearance using allometric dose scalers (e.g., Cl scaled by ABW to a power of “*x*”) [46–48]. These models, however, have not been well-validated particularly in critically ill obese patients and are not commonly used in the clinical setting. Furthermore, the inherent differences in the endpoints targeted with each strategy (i.e., induction of anesthesia for a surgery vs. mild-to-moderate sedation for comfort) are noteworthy making the extrapolation of these models limited.

The most common and often dose-limiting adverse effect with propofol is hypotension. One study identified obesity as a risk factor for hypotension in trauma patients who received propofol in the emergency department [49]. In this study, obesity (BMI ≥ 30 kg/m^2^) was associated with more than a 2-fold increase in hypotension [OR (95% CI) = 2.66(1.08–6.55)]. This is likely related to the higher doses administered or the use of ABW-based dosing in the obese cohort.

##### Summary

Although some studies support the use of ABW-based dosing for propofol, the relationship between weight and pharmacokinetic variables such as clearance is nonlinear. Dosing using ABW may result in supratherapeutic concentrations. Thus, weight-based dosing using either IBW or adjusted body weight is preferred.

#### Dexmedetomidine

Pharmacokinetic studies of dexmedetomidine in critically ill patients have revealed a volume of distribution of 104 L and clearance of 39 L/h with a substantial amount of interpatient variability [50]. Hypoalbuminemia, end-organ damage, and cardiac output may contribute to this variability [51]. The log *P* for dexmedetomidine is 3.39.

Studies evaluating the impact of obesity on dexmedetomidine pharmacokinetics are beginning to emerge but data specific to the ICU population are limited (Additional file 2). Shortcomings related to the study setting (i.e., operating room versus ICU) would apply. One study evaluated the pharmacokinetics of dexmedetomidine in morbidly obese patients undergoing laparoscopic surgery [52]. There was no difference in volume of distribution when normalized to ABW (2.48 ± 0.47 vs. 2.38 ± 0.72 L/kg; *p* = .72) but clearance was significantly lower with obesity (0.47 ± 0.07 vs. 0.64 ± 0.09 L/kg/h; p < .01, based on ABW). As a result, area under the curve was significantly larger (2174 ± 335 vs. 1594 ± 251 ng L/h, *p* < .01). A second study, also conducted in the operating room setting, revealed dexmedetomidine dosing based on a linear ABW-based strategy led to higher serum concentrations in obese compared to non-obese patients [53]. This was presumed to be due to both decreased distribution into adipose tissue and impaired clearance associated with fat mass. A follow-up study by the same research team however concluded fat mass did not influence dexmedetomidine clearance [54]. Nevertheless, lean body weight was the preferred weight measure for dosing.

##### Summary

The use of ABW to calculate either bolus doses or infusion rates may lead to supratherapeutic concentrations. Weight-based dosing for dexmedetomidine using either IBW or adjusted body weight is suggested.

#### Benzodiazepines

Midazolam is a highly lipophilic benzodiazepine (log *P* = 3.33) with a volume of distribution of 2 L/kg. Initial studies in healthy volunteers demonstrated a significantly higher volume of distribution, even after controlling for ABW, in obese subjects (1.74 ± 0.11 vs. 2.66 ± 0.16 L/kg; *p* < .001) [55] (Additional file 2). Total clearance, however, was not impacted by obesity (non-obese, 530 ± 34 vs. obese, 472 ± 38 ml/min; *p* = NS). Overall, this led to a prolonged half-life with obesity (2.27 ± 0.3 vs. 5.94 ± 0.85 h; *p* < .001). A second study evaluated midazolam pharmacokinetics in obese patients undergoing bariatric surgery [56]. In this study, there was a linear increase in central volume of distribution with increased ABW and peripheral volume of distribution increased in a non-linear manner. Clearance was unaffected by ABW.

##### Summary

The data suggest larger initial doses may be necessary in obese patients because of the increased volume of distribution observed in these patients. Midazolam clearance however does not change with increasing ABW thus the potential for accumulation and supratherapeutic concentrations exists with weight-based dosing using ABW. Because of concerns with adverse hemodynamic effects with larger doses of midazolam, a safer approach would be to use IBW or adjusted body weight for initial doses with smaller supplemental doses administered as needed until the desired effect is achieved. Adjusted body weight or IBW should be used to calculate doses for continuous infusions.

#### Etomidate

Most of the recent research concerning the use of etomidate in critically ill patients involves the administration of single intravenous bolus doses for rapid sequence intubation (RSI). Irrespective of the favorable hemodynamic adverse effect profile of etomidate in critically ill patients, there is ongoing debate of the use of etomidate for RSI because of potential adrenal insufficiency related to inhibition of 11 beta-hydroxylase [57, 58]. Despite these concerns, etomidate continues to be used and investigated as an agent for RSI with a usual dose of 0.3 mg/kg based on ABW [59, 60]. Etomidate has a large volume of distribution with relatively rapid metabolism by the liver, but with plasma concentrations that are poorly correlated with pharmacodynamic measures of clinical response [61]. In light of etomidate’s large volume of distribution, there is a concern of under-dosing in more obese patients, which could lead to patient awareness during concomitant paralysis with a neuromuscular blocker [62]. In one prospective study evaluating an RSI protocol, 5 of the 10 patients interviewed remembered aspects of the intubation procedure suggesting inadequate sedation [5]. Concerns of inadequate dosing would outweigh toxicity concerns of higher doses based on ABW in most obese patients. However, there are practical concerns related to etomidate vial and syringe sizes available in bedside RSI kits that may hinder timely administration of large etomidate doses based on ABW. Re-dosing of etomidate postintubation is another important consideration since the duration of action of etomidate is substantially shorter than that of commonly used nondepolarizing neuromuscular blockers such as rocuronium [63].

##### Summary

For dosing etomidate, ABW is suggested for weight-based dosing in obese patients with a BMI < 40 kg/m^2^. Dosing using either adjusted or ABW is suggested in patients with more severe forms of obesity (e.g., BMI ≥ 40 kg/m^2^).

### Antipsychotics

#### Haloperidol

Despite the limited role antipsychotics have in evidence-based guidelines for both the prevention and treatment of delirium, they are still widely administered for this indication [3, 64, 65]. Haloperidol has a volume of distribution of 1260 L and a log *P* of 3.77 suggesting distribution into adipose tissue [66]. One study evaluating haloperidol pharmacokinetics in psychiatric patients reported a non-linear relationship between body weight and clearance [67]. Nevertheless, a troublesome adverse effect with haloperidol is QTc prolongation, which can be associated with dose. Further, a higher incidence of torsades de pointes has been found in patients who receive at least 35 mg of haloperidol in 24 h [68]. The incidence of torsades de pointes was more prominent when this dose was given in less than 6 h. Caution with larger doses is therefore warranted.

##### Summary

Although haloperidol has pharmacokinetic properties that favor distribution into adipose tissue, there are severe adverse reactions associated with large doses. Standard doses of haloperidol that can be titrated to effect are suggested. Routine monitoring for QTc prolongation should be conducted.

#### Quetiapine

Quetiapine is an atypical antipsychotic frequently considered in place of haloperidol because of a more favorable adverse effect profile. Quetiapine also has a large volume of distribution (10 L/kg) with a log *P* of 2.81. There are no data evaluating quetiapine dosing in obese critically ill patients. Pharmacokinetic studies in healthy volunteers or individuals with psychiatric disorders have not reported significant variance secondary to weight [69]. Few, if any of these patients however would be expected to have extreme forms of obesity.

##### Summary

Standard, non-weight-based doses of quetiapine consistent with that utilized in non-obese patients should be considered.

## Conclusion

For obese patients, there is no high-level clinical evidence available to help design dosing regimens for sedation, analgesia, and delirium as recommended in critical care practice guidelines. Based on pharmacokinetic studies, the relationship between ABW and pharmacokinetic variables such as volume of distribution and clearance is not linear for many medications used in the management of pain, agitation, and delirium. For such medications, standard, non-weight-based dosing, or weight-based dosing using either IBW or adjusted body weight, is appropriate. Weight-based dosing using ABW is discouraged because dose proportionality between pharmacokinetic parameters and ABW is rarely encountered. In the rare instances where dose proportionality is evident, potential adverse effects (associated with large doses) remain an important consideration. Clinicians should utilize smaller doses that can be repeated incrementally and titrated to clinical effect when applicable. Consultation with a clinical pharmacist can be useful when crafting dosing regimens in critically ill patients with extreme obesity.

## Supplementary information

**Additional file 1.** Hypothetical examples of initial doses in three male patients with different weights using ideal body weight, adjusted body weight and actual body weight. For each example, height is estimated to be 5’9” and adjusted body weight is calculated using a correction factor of 0.4.

**Additional file 2.** Clinical and pharmacokinetic studies involving sedatives.

## Data Availability

Not applicable

## Abbreviations

ABW: Actual body weight
BMI: Body mass index
BSA: Body surface area
IBW: Ideal body weight
ICU: Intensive care unit
