# Supplementary material for: Drug dosing in the critically ill obese patient—a focus on sedation, analgesia, and delirium
Source: Crit Care. 2020 Jun 8;24:315. doi: 10.1186/s13054-020-03040-z (PMC7282067; doi:10.1186/s13054-020-03040-z)
Supplement: Supplementary file 1 — Additional file 1. Hypothetical examples of initial doses in three male patients with different weights using ideal body weight, adjusted body weight and actual body weight. For each example, height is estimated to be 5’9” and adjusted body weight is calculated using a correction factor of 0.4. [file 13054_2020_3040_MOESM1_ESM.docx]

**Additional File 1. Hypothetical examples of initial doses in three male patients with different weights using ideal body weight, adjusted body weight and actual body weight. For each example, height is estimated to be 5’9” and adjusted body weight is calculated using a correction factor of 0.4.**

| **Medication** | **Weight-based Starting Dose** | **Weight Metric for Calculations** | **70 kg Patient** | **100 kg Patient** | **140 kg Patient** |
| --- | --- | --- | --- | --- | --- |
| Propofol | 5 mcg/kg/min | **IBW** | 350 mcg/min | 350 mcg/min | 350 mcg/min |
|  |  | **AdjBW** | 350 mcg/min | 410 mcg/min | 490 mcg/min |
|  |  | **ABW** | 350 mcg/min | 500 mcg/min | 700 mcg/min |
| Dexmedetomidine | 1 mcg/kg, then  0.2 mcg/kg/hr | **IBW** | 70 mcg, then 14 mcg/hr | 70 mcg, then 14 mcg/hr | 70 mcg, then 14 mcg/hr |
|  |  | **AdjBW** | 70 mcg, then 14 mcg/hr | 82 mcg, then 16.4 mcg/hr | 98 mcg, then 19.6 mcg/hr |
|  |  | **ABW** | 70 mcg, then 14 mcg/hr | 100 mcg, then 20 mcg/hr | 140 mcg, then 28 mcg/hr |
| Midazolam | 0.05 mg/kg, then 0.02 mg/kg/hr | **IBW** | 3.5 mg, then 1.4 mg/hr | 3.5 mg, then 1.4 mg/hr | 3.5 mg, then 1.4 mg/hr |
|  |  | **AdjBW** | 3.5 mg, then 1.4 mg/hr | 4.1 mg, then 1.64 mg/hr | 4.9 mg, then 1.96 mg/hr |
|  |  | **ABW** | 3.5 mg, then 1.4 mg/hr | 5 mg, then  2 mg/hr | 7 mg, then  2.8 mg/hr |
| Fentanyl | 0.7 mcg/kg/hr | **IBW** | 49 mcg/hr | 49 mcg/hr | 49 mcg/hr |
|  |  | **AdjBW** | 49 mcg/hr | 57 mcg/hr | 68.6 mcg/hr |
|  |  | **ABW** | 49 mcg/hr | 70 mcg/hr | 98 mcg/hr |
| Etomidate | 0.3 mg/kg | **IBW** | 21 mg | 21 mg | 21 mg |
|  |  | **AdjBW** | 21 mg | 24.6 mg | 29.4 mg |
|  |  | **ABW** | 21 mg | 30 mg | 42 mg |
| Ketamine | 0.5 mg/kg, then  1 mcg/kg/min | **IBW** | 35 mg, then  70 mcg/min | 35 mg, then  70 mcg/min | 35 mg, then  70 mcg/min |
|  |  | **AdjBW** | 35 mg, then  70 mcg/min | 41 mg, then  82 mcg/min | 49 mg, then  98 mcg/min |
|  |  | **ABW** | 35 mg, then  70 mcg/min | 50 mg, then 100 mcg/min | 70 mg, then 140 mcg/min |

IBW = ideal body weight; AdjBW = adjusted body weight; ABW = actual body weight
