# Supplementary material for: Drug dosing in the critically ill obese patient—a focus on sedation, analgesia, and delirium
Source: Crit Care. 2020 Jun 8;24:315. doi: 10.1186/s13054-020-03040-z (PMC7282067; doi:10.1186/s13054-020-03040-z)
Supplement: Supplementary file 2 — Additional file 2. Clinical and pharmacokinetic studies involving sedatives. [file 13054_2020_3040_MOESM2_ESM.docx]

**Additional File 2.** Clinical and pharmacokinetic studies involving sedatives

| Reference | Treatment | Control | Results | Comments |
| --- | --- | --- | --- | --- |
| *Propofol* | | | | |
| Leslie, 1991 (41) | N = 92 patients with a mean weight of 73 ± 11 kg for men and 65 ± 10 kg for females receiving propofol for induction of anesthesia. Doses were based on lean tissue mass. | No placebo or control | The mean ED50 for males was 1.31 mg/kg versus 1.28 mg/kg for females (p=NS). | Based on average weight, there were few patients with obesity.  Authors concluded that because there was no difference in dosing requirements based on lean tissue mass, despite differences in body habitus between males and females, lean tissue mass would be the preferred weight metric. |
| Servin, 1993 (36) | N= 8 patients with a mean weight of 116 ± 21 kg receiving propofol for induction and maintenance of anesthesia | N = 10 non-obese patients who weighed 66 ± 15 kg obtained from a concurrent study of propofol pharmacokinetics | Both clearance and volume of distribution were correlated to actual body weight (clearance, R = 0.76; volume of distribution, R = 0.61) | Mean volume of distribution was obese and non-obese patients was 17.9 ± 28.2 L and 13 ± 7.4 L (p=NS); mean clearance for obese and non-obese patients was 24.3 ± 6.2 and 28.3 ± 6.6 ml/min/kg (p=NS). |
| Lind, 1993 (42) | N = 177 patients receiving propofol for induction and maintenance of anesthesia. | No placebo or control | Extubation time was correlated to body mass distribution; non-abdominal distribution of body mass was associated with shorter extubation times. | Weight range was not reported |
| Chassard, 1996 (38) | N = 21 female patients with a mean weight of 55 kg who received propofol for induction of anesthesia. | No placebo or control | Propofol dosing requirements was not proportional to weight but was related to lean body mass (determined by BIA) and BMI. | Range of patient weights did not exceed 88 kg. |
| Hirota, 1999 (37) | N = 60 patients with a mean weight of 58 ± 13 kg receiving propofol for induction and maintenance of anesthesia | No placebo or control | There was a significant correlation between propofol concentration and actual body weight (R=0.646, P<.001). | Only 1 patient had a BMI > 31 kg/m^2^. |
| McLeay, 2009 (43) | Simulated dosing model in subjects weighing between 70 and 160 kg that included a linear relationship between clearance and lean body weight. | A prior dosing model that included a nonlinear relationship between clearance and actual body weight. | Dosing using actual body weight resulted in increasing plasma concentrations as patient weight increased. Dosing using lean body weight resulted in similar profiles across all weights. | Dosing using actual body weight suggested an increased time to awakening in the 160 kg subjects versus the 70 kg subjects (77% vs. 42%). |
| Cortinez, 2010 (46) | N = 19 patients with a mean weight of 106 ± 18 kg who received propofol for induction and maintenance of anesthesia. These results were also pooled with patients from previous studies (total N = 51, weight = 93 ± 24 kg) | No placebo or control | Index patients: A linear model using actual body weight was superior to other size descriptors. | Pooled patients: An allometric model using actual body weight with an exponent of 0.75 was superior to the linear TBW model |
| Ingrande, 2011 (40) | N = 60 patients who were randomized to receive propofol for induction of anesthesia based on actual body weight (N=30, mean weight = 133 kg) or lean body weight (N=30, mean weight = 130 kg) | N = 30 non-obese control patients with a mean weight of 60 kg. | The relationship between lean body weight and dose was stronger than the relationship between actual body weight and dose for both obese (R^2^ = 0.74 vs. 0.65) and non-obese patients (R^2^ = 0.58 vs. 0.49). | Time to loss of consciousness was 65, 94 and 86 seconds for the actual body weight, lean body weight and non-obese groups, respectively. |
| Van Kralingen, 2011 (48) | N = 20 patients with a mean weight of 124 ± 20 kg who received propofol for induction and maintenance of anesthesia | N = 44 non-obese patients with a mean weight of 74 ± 11 kg from previously published studies. | Actual body weight using an allometric function with an exponent of 0.72 was the best determinant of clearance in obese patients. | Weight was not a covariate for pharmacodynamic effects, namely depth of sedation via BIS. |
| Lam, 2013 (45) | N = 18 patients with a mean weight of 109 ± 20 kg who received propofol for induction of anesthesia dosed according to actual body weight. | N = 20 patients with a mean weight of 112 ± 22 kg who were dosed using a corrected body weight formula with a 60% adjustment factor.[lean body weight + (actual body weight – lean body weight) x 60%] | Lower doses were noted in the patients dosed according to corrected body weight but there were no differences in the highest BIS value or in blood pressure. | This was a randomized controlled trial. |
| Eleveld, 2014 (47) | Aggregate of 21 previously published studies representing 660 individuals with a weight range of 5.2 to 160 kg. | No placebo or control | Allometric scaling exponent of 0.75 for clearance performed well. Volume of the central compartment was independent of weight for weights > 30 kg. | There were 36 individuals identified as “high-BMI”. Poorer predictive performance of the model was noted in these individuals. |
| Dong, 2016 (39) | N = 23 patients with a BMI > 35 kg/m^2^ were randomized to receive propofol for anesthesia induction based on actual body weight (N=12) or lean body weight (N=11) | N = 6 non-obese control patients with a BMI < 25 kg/m^2^ who received propofol based on actual body weight | Higher clearance values were noted in the patients with obesity (actual body weight group, 10 L/min; lean body weight group, 9.15 L/min vs. non-obese, 4.11 L/min, p<.01). Volume of the peripheral compartment was higher with obesity (actual body weight group, 73.2 L; lean body weight group, 84.2 L vs. non-obese, 46.9 L, p<.01). | This was a prospective randomized controlled trial.  BIS values were significantly lower in the obese patients who were dosed using actual body weight. |
| Araujo, 2018 (44) | N = 20 patients with a mean BMI of 42.6 ± 4.8 kg/m^2^ who received propofol for induction of anesthesia | N = 20 non-obese patients with a mean BMI of 25.4 ± 3.4 kg/m^2^ | Propofol concentrations were higher in the obese group (4.9 ± 1 vs. 3.8 ± 1.5 mcg/ml, p<.012). A poor correlation was observed between dose and all weight metrics evaluated (i.e., actual body weight, fat free mas and fat mass) | Higher propofol concentrations suggest volume of distribution in the central compartment is not increased in obesity. |
| *Dexmedetomidine* | | | | |
| Valitalo, 2013 (50) | N = 527 critically ill patients with a mean weight of 80 ± 20 kg who were enrolled in 3 previously conducted clinical trials | No placebo or control | Positive correlation between body weight and clearance. Scaling exponent for weight in final model was 0.76 | Analysis included predominantly non-obese individuals. Correlation indicates a weight-based approach for dosing (vs. a fixed dose) is appropriate. |
| Cortinez, 2015 (53) | N = 20 obese patients with a mean weight of 115 kg who received dexmedetomidine dosed according to actual body weight in the operating room | N = 20 non-obese patients with a mean weight of 75 kg | A linear actual body weight size model was associated with higher serum concentrations than in the non-obese cohort. | Fat free mass better characterized pharmacokinetic changes in the obese. |
| Xu, 2017 (52) | N = 8 obese patients with a mean weight of 125 ± 10 kg who received dexmedetomidine in the operating room | N = 8 non-obese patients with a mean weight of 70 ± 6 kg | Obese patients had a higher volume of distribution (310 ± 63 vs. 164 ± 41 L, p<.001) and clearance (59 ± 11 vs. 45 ± 9 L/hr, p=.02). Cmax (3.75 ± 0.56 vs. 2.54 ± 0.32 mcg/L, p<.001) and AUC (2174 ± 335 vs. 1594 ± 251 ng hr/L) were significantly higher in the obese cohort. | When normalized to actual body weight, there was no difference in volume of distribution and clearance was significantly lower in morbidly obese patients. |
| Rolle, 2018 (54) | N = 40 patients with a mean weight of 90 kg (range, 47 – 126 kg) who received dexmedetomidine in the operating room | No placebo or control | Final pharmacokinetic model included volume of distribution and clearance scaled linearly to lean body weight. | Fat mass did not impact clearance |
| *Midazolam* | | | | |
| Greenblatt, 1984 (55) | N = 20 healthy, obese volunteers with a mean weight of 117 ± 8 kg who received a single dose of midazolam 2.5 – 5 mg IV on one occurrence and 5 – 10 mg orally on another | N = 20 healthy, non-obese patients with a mean weight of 66 ± 1.5 kg | The volume of distribution was higher in the obese cohort (311 ± 27 vs. 114 ± 7 L, p<.001). Clearance in the obese group was 472 ± 38 ml/min vs. 530 ± 34 ml/min in the non-obese group (p=NS) | Elimination half-life was significantly longer in the obese group (8.4 ± 0.84 vs. 2.73 ± 0.34 hours, p<.001) |
| Brill, 2014 (56) | N = 20 obese patients with a mean weight of 144 ± 22 kg who received midazolam 7.5 mg orally followed by 5 mg IV prior to bariatric surgery. | N = 12 healthy volunteers with a mean weight of 76 ± 8.7 kg | Central volume of distribution increased linearly with body weight. Clearance was not influenced by weight. | Increased half-life was noted in obesity. |

BMI = body mass index; BIA = bioimpedance analysis ; ED50 = median effective dose; BIS = bispectral index; Cmax = maximum concentration; AUC = area under the curve; TBW = total body weight
